# 新型纳米材料在农产品安全分析中的应用进展

**DOI:** 10.3724/SP.J.1123.2022.09010

**Published:** 2023-09-08

**Authors:** Ranfeng ZHOU, Huixian ZHANG, Xiaoli YIN, Xitian PENG

**Affiliations:** 1.湖北省农业科学院农业质量标准与检测技术研究所, 农产品营养品质与安全湖北省重点实验室, 湖北 武汉 430064; 1. Institute of Agricultural Quality Standards and Testing Technology Research, Hubei Academy of Agricultural Science, Hubei Key Laboratory of Nutritional Quality and Safety of Agro Products, Wuhan 430064, China; 2.长江大学生命科学学院, 湖北 荆州 434023; 2. College of Life Science, Yangtze University, Jingzhou 434023, China

**Keywords:** 新型纳米材料, 农产品质量安全, 样品制备, 综述, novel nanomaterial, quality safety of agricultural products, sample preparation, review

## Abstract

农产品质量安全事关民生福祉,近年来受到了政府和消费者越来越多的关注,建立农产品中农药、兽药、重金属和真菌毒素等污染物高效、快速和灵敏的分离分析新方法,对于保障农产品质量安全具有重要的意义。农产品基质复杂,污染物浓度低,采取适当的样品前处理对农产品中的污染物进行富集净化是非常重要的。固相萃取是目前应用最多的样品前处理技术,其核心吸附剂决定了萃取的选择性和萃取效率。近年来,越来越多的新型材料被用作固相萃取的吸附剂,结合多种萃取模式(固相微萃取、分散固相萃取、磁性固相萃取等),大大提高了目标物的萃取效率、萃取选择性和分析通量。纳米材料具有大的比表面积,对痕量目标物亲和力强,将其作为固相萃取的吸附剂,极大地改善了分析技术的选择性和灵敏度,已经成为农产品中痕量化合物预富集技术的优先选择。本文概述了磁性材料、碳基材料、金属和金属氧化物材料、金属有机骨架材料、有机共价骨架材料等纳米材料的吸附特性,因具有比表面积大、吸附容量高、结构可设计等众多优点,良好的稳定性和优异的物理化学性能使其非常适合作为农产品安全分析中污染物富集净化的吸附剂,结合色谱、光谱、质谱等检测技术,所开发的分析方法成功应用于多种农产品中污染物的分析,很好地消除了基质干扰,提高了样品前处理的萃取效率、选择性和分析通量。本文围绕几类新型纳米分离材料在农产品质量安全分析中的应用进行了综述,重点阐述了吸附剂与目标物的作用机理,并对其进一步的开发和应用进行了展望,以期为新型纳米分离介质的制备及其在农产品安全分析中的应用提供参考。

## Abstract (en)

The quality and safety of agricultural products are strongly related to human livelihood. Thus, the government and consumers have recently paid increased attention to the quality and safety of agricultural products. The development of efficient, rapid, and sensitive analytical methods for detecting pesticides, veterinary drugs, heavy metals, mycotoxins, and environmental pollutants in agricultural products is of great significance. Owing to the complexity of many sample matrices and the low concentration of pollutants in a typical sample, appropriate sample pretreatment steps are necessary to enrich pollutants in agricultural products. Solid-phase extraction (SPE) is the most widely used sample pretreatment technology; in this technique, the adsorbent generally determines the selectivity and efficiency of the extraction process. An increasing number of novel materials have been used as SPE adsorbents. The extraction efficiency, extraction selectivity, and analytical throughput of SPE could be greatly improved by combining these novel materials with various extraction modes (e. g., solid-phase microextraction, dispersed SPE, and magnetic SPE (MSPE)) during sample preparation. Because of their large specific surface area and high affinity toward target analytes, nanomaterials are often used as SPE adsorbents, thereby greatly improving the selectivity and sensitivity of the analytical technology. More importantly, these materials have become a priority area of research on preconcentration technologies for trace compounds in agricultural products. This paper summarizes the adsorption characteristics of several new nanomaterials, including magnetic materials, carbon-based materials, metal nanomaterials (MNs), metal oxide nanomaterials (MONs), metal organic frameworks (MOFs), and covalent organic frameworks (COFs). These nanomaterials present numerous advantages, such as large specific surface areas, high adsorption capacities, and tailorable structural designs. MSPE employs magnetic materials as sorbents to afford fast dispersion and efficient recycling when applied to complex sample matrices under an external magnetic field. The use of MSPE can avoid several typical problems associated with SPE such as poor adsorbent packing and high pressure, thereby greatly simplifying the pretreatment process and providing a high flux for sample analysis. Carbon-based materials are powdered or bulk nonmetallic solid materials with carbon as the main component; carbon and nitrogen materials, mesoporous carbon, carbon nanotubes, and graphene are some examples of these materials. These materials provide large specific surface areas, abundant pore structures, good thermal stability, high mechanical strength and adsorption capacity, and controllable morphology. Pure and modified carbon nanomaterials have been successfully used to purify target analytes from agricultural products. Given their unique physical and chemical properties, MNs and MONs have attracted significant interest for use in sample preparation. MNs and MONs with excellent thermal and mechanical stabilities show good resistance to a wide pH range and diverse organic solvents, which is crucial in adsorbent-based extraction methods. The surface of these materials can be easily modified with various ligands to improve their selectivity. MOFs and COFs present many advantages such as large specific surface areas, high porosity, adjustable pore performance, and good thermal stability. Several methods that employ novel adsorbent materials to analyze pollutants in a variety of agricultural products, such as chromatography, spectroscopy, mass spectrometry, and other detection technologies, have been established. This paper also reviews the application of adsorbent materials in the analysis of agricultural product quality and safety, and discusses the future development trends of these sorbents in sample preparation for the safety analysis of agricultural products.

随着人们生活水平的提高,食品安全越来越受到人们的重视,农产品质量安全是食品安全的基础,对农产品中污染物进行高效快速的分析对于保障农产品质量安全具有重要的意义。农产品中污染物种类很多,例如农药、兽药、重金属、真菌毒素、违禁食品添加剂等^[1]^。一般来说,大部分污染物处于痕量水平,同时样品基质复杂,使用现有仪器直接检测非常困难,在仪器分析前进行适当的样品前处理对农产品中的污染物进行提取净化十分重要。

传统的样品制备方法例如固相萃取(SPE)、液液萃取(LLE)、凝胶渗透色谱(GPC)^[2⇓⇓-5]^在农产品安全分析中取得了一些进展,但其存在自动化困难、时间长、溶剂消耗量大等缺点。随着样品分析通量和环境保护的要求越来越高,这些传统的前处理方法已经不能很好地满足样品分析的要求。因此,有必要开发高选择性、快速、简便、准确的样品前处理方法,以满足农产品安全分析的需求。在各种样品前处理方法中,SPE是目前应用非常广泛的样品前处理技术,其核心是吸附剂,吸附剂的选择决定了萃取的选择性和萃取效率。传统的反相C18和正相硅胶SPE吸附剂选择性较差,吸附容量不高,新型高效的吸附剂亟待开发。纳米材料(NMs)是指在颗粒或孔隙至少一个维度上具有纳米尺度(通常在1~100 nm)的一种特殊材料。与传统的微米或更大尺寸的材料相比,纳米尺寸的结构使得材料具有一些特殊的性质。由于大的比表面积、对痕量目标物高的亲和力,纳米材料作为固相萃取的吸附剂,极大地改善了分析技术的选择性和灵敏度,已经成为农产品中痕量化合物预富集技术的优先选择。近年来一些新型的纳米材料例如碳基材料、磁性材料、金属有机骨架材料、金属氧化物等被作为固相萃取的吸附剂,结合高效的样品前处理技术,在农产品安全分析中取得了较好的效果(图1)。本文对近年来一些新型分离材料在农产品安全分析中的应用进行了综述,重点对纳米吸附剂与目标物的作用机理进行了分析,以期为新型纳米材料在农产品安全分析中的应用提供参考。

**图 1:**
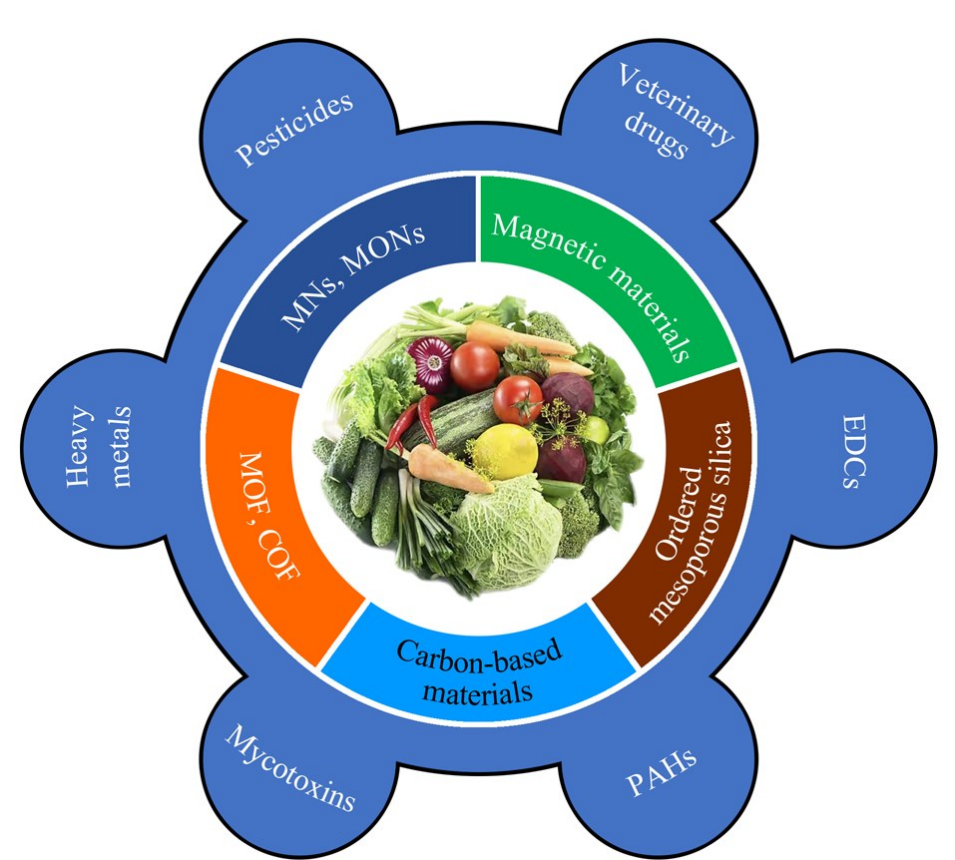
新型纳米材料在农产品安全分析中的应用

## 1 农产品样品前处理中的纳米材料

### 1.1 磁性纳米材料

四氧化三铁(Fe_3_O_4_)是目前应用最广泛的磁性分离材料,具有高饱和磁化率及超顺磁性特点,其具有比表面积大、易于功能化、环境友好等诸多优点。将其作为磁性固相萃取(MSPE)的吸附剂,在外加磁场作用下,吸附剂和样品溶液能够实现快速分离,避免了离心、过滤等复杂操作和填料层堵塞等典型问题,大大简化了前处理过程,提高了样品分析的通量^[6]^。同时,分散固相萃取的模式可以实现与样品溶液与吸附剂充分接触,加快了吸附平衡,提高了萃取效率。我们课题组直接将未修饰的磁性Fe_3_O_4_纳米粒子应用于食用油中黄曲霉毒素(AFs)和阿维菌素的分析,食用油样品经过简单的稀释后,吸附剂通过氢键等极性相互作用,可以直接从食用油样品中萃取黄曲霉毒素和阿维菌素^[7,8]^。然而,纯的磁性材料萃取选择性还存在一定的局限,在萃取过程中容易发生团聚,为了克服这些问题,对其进行适当的改性是非常必要的。近年来,各种功能基团和新型材料,例如有机配体^[9]^、聚合物^[10]^、离子液体^[11]^、碳材料^[12]^等都用于Fe_3_O_4_的修饰,在农产品中农药、兽药、真菌毒素、重金属等的分析中取得了很好的效果。这些功能化的磁性吸附剂一方面保留磁性吸附剂快速相分离的优势,另一方面针对农产品中不同的污染物设计合适的官能团修饰,大大提高吸附剂的选择性和萃取效率^[13]^。

### 1.2 碳基纳米材料

碳基纳米材料如碳纳米管(CNTs)、石墨烯(G)、石墨烯氧化物(GO)、氮化碳纳米材料(CNNs)等,具有比表面积大、吸附容量高、化学及物理稳定性好等优点,近年来作为农产品质量安全分析前处理过程的吸附剂,取得了很好的应用效果^[14]^。CNTs是碳的同素异形体,CNTs可分为单壁碳纳米管(SWCNTs)和多壁碳纳米管(MWCNTs), SWCNTs由单片石墨烯制成,而MWCNTs由多个石墨烯片组成,其比表面积最高可达1500 m^2^/g^[15]^,因而CNTs吸附能力强、吸附容量高。我们课题组采用物理混合的方法制备了一种磁性的羧基官能化的MWCNTs,不同于传统的化学修饰方法,直接将氨基官能化的MWCNTs和Fe_3_O_4_在溶剂中进行混合组装,可以将非磁性氨基官能化的MWCNTs转化为磁性吸附剂,利用吸附剂与苯氧乙酸类农药之间的离子交换、*π-π*和疏水相互作用,成功地应用于环境水中苯氧乙酸类农药的快速富集净化^[16]^。G是一种新型二维碳纳米材料,具有高比表面积和大的*π*电子共轭体系,以及良好的稳定性和优异的物理化学性能,是样品制备中提取和富集芳环结构目标物的理想材料^[17]^。然而,G的疏水性太强,在水溶液中不易分散,影响了其萃取性能。GO通过将G采用合适的氧化剂氧化而来,其表面含有羧基、羰基或羟基等含氧官能团,可以显著提高材料的亲水性,同时其特殊的分子结构可以与目标物分子发生强的电荷转移和*π-π*相互作用,从而实现农产品中痕量污染物快速高效的富集净化^[18]^。此外,由三对三嗪单元组成的CNNs作为固相萃取剂在农产品安全分析中的应用也受到越来越多的关注,其结构中含有丰富的氮官能团和较大的离域*π*键,因此CNNs可以与目标物发生化学络合、离子交换、强*π-π*共轭、氢键和静电等多种相互作用,显著改善萃取介质的选择性和萃取能力,有效地萃取净化农产品农药^[19]^、兽药^[20,21]^、多环芳烃^[22]^等污染物。

### 1.3 金属和金属氧化物纳米材料

金属纳米材料(MNs)和金属氧化物纳米材料(MONs)由于其独特的物理和化学性质,在样品制备方面引起了人们的极大兴趣。相比于有机聚合物吸附材料,MNs或MONs具有更好的热稳定性和机械稳定性,宽pH范围和对多种有机溶剂具有良好的耐受性,在极端条件下可以获得好的吸附和解吸性能。MNs或MONs表面容易修饰各种配体,其可以根据具体的目标物设计合适的功能团,大大提高了吸附剂的选择性。MNs在农产品安全分析中应用最多的是金纳米粒子(AuNPs)和银纳米粒子(AgNPs),它们具有尺寸可控、化学惰性、比表面积大、生物相容性好、表面修饰简单等独特的性能。与MNs相比,MONs在样品制备中的应用更广泛,这类材料包括TiO_2_、ZrO_2_、Al_2_O_3_等。一方面,MONs可以利用其特殊的性质,直接作为样品前处理的吸附剂材料,例如利用氧化镁表面的氧孔穴和*π*电子相互作用可以用于萃取农产品中的多环芳烃^[23]^, ZrO_2_表面的路易斯酸位点可以用于除去农产品中脂类和羧酸类的干扰物^[24]^,部分重金属氧化物可以直接吸附样品中的重金属离子等,*γ*-Al_2_O_3_用于分析农产品中的重金属^[25]^、农药^[26]^等污染物。另一方面,MONs还可以进行适当的官能化,进一步提高吸附剂的选择性,例如将金属氧化物与其他功能无机材料(磁核^[27]^、碳纳米材料^[28]^、介孔SiO_2_^[29]^等)结合,使吸附剂除了具有MONs的性质外,还具有其他独特的功能特性,从而极大地促进了其在样品制备中的应用。近期,我们课题组介绍了基于MNs和MONs样品制备的最新成果及研究进展,分析了用于样品制备中MNs和MONs的性质及其与目标物的作用机理,为MNs和MONs在农产品安全分析中的应用提供理论指导^[30]^。

### 1.4 金属有机骨架和有机共价骨架纳米材料

金属有机骨架(MOFs)是一种由无机金属离子(或金属簇)作为节点或者中心,与有机配体通过自组装的方式形成的具有周期性和无限延伸骨架结构的多孔晶体材料,具有比表面积大、金属位点开放、空隙率高和较好的物理化学稳定性等优点^[31]^,广泛应用于催化^[32,33]^、吸附和分离^[34]^、传感^[35]^、生物医学^[36,37]^等领域。与MOFs相比,通过轻元素(C、H、B、N、O等)的可逆共价键合成的有机共价骨架(COFs)也表现出类似的优点,密度低,结构可调,易于功能化等^[38]^。MOFs和COFs具有独特的微观结构以及优异的性能,都是极具应用前景的吸附剂。一方面,MOFs和COFs多孔的结构和大的共轭体系能够提供大量*π-π*相互作用位点,同时其官能团的修饰还可以进一步改善萃取的选择性,可以直接用作多种微固相萃取的吸附剂。我们课题组一步法合成了氨基官能化的MIL-101(Fe)和MIL-101(Cr),分别用于分散固相萃取和注射泵辅助微固相萃取的吸附剂,成功应用于农产品中苯氧乙酸类农药和有机磷类农药的快速提取净化^[39,40]^。另一方面,为了解决MOFs和COFs稳定性的问题,采用合适的方法制备的MOFs(COFs)和碳材料^[41]^、磁性材料^[42]^、聚合物^[43]^等的复合材料,结合了多种功能材料的优势,在农产品中多环芳烃、农药、重金属等富集净化中取得了很好的效果。其中,基于MOFs和COFs的磁-壳结构纳米材料近年来引起了科研人员的广泛关注,其结合了磁吸附剂的快速分离和MOFs(COFs)良好的吸附性能,在农产品安全分析中具有很好的应用潜能。

### 1.5 有序介孔硅胶纳米材料

有序介孔硅胶(OMS)是以表面活性剂的超分子组装体为结构导引试剂,硅源在酸性或碱性条件下水解而获得的一类多孔固体,最常见的有MCM-41、MCM-48和SBA-15。有序介孔硅胶纳米材料由于其高度有序的介孔结构、大的比表面积、可调节的孔结构以及易于化学修饰等特点,在催化反应、电化学、光学传感器、纳米粒子合成、样品前处理等方面应用非常广泛。在样品前处理方面,有序介孔硅胶可以直接作为固相萃取的吸附剂,也可以通过一定的官能团修饰,提高吸附剂的选择性,用于农产品中农药、重金属、真菌毒素等污染物的分析^[44]^。此外,磁性有序介孔硅胶纳米材料结合了介孔硅胶的形貌特征和磁分离特征,表面易于修饰,在样品前处理中具有很好的应用前景。我们课题组采用伪晶转化的方法将非介孔结构的磁性二氧化硅转化为有序介孔的磁性二氧化硅,将其直接用于食用油中有机磷类农药的萃取,介孔的结构可以排斥大分子的干扰,而硅羟基可以与有机磷类农药发生氢键等相互作用,实现食用油中有机磷类农药快速有效的萃取净化^[45]^。同时,为了进一步提高吸附剂的选择性,在磁性介孔硅胶表面键合了辛基和氨基官能团,通过离子交换和疏水相互作用,实现了苯氧乙酸类农药高选择性的富集^[46]^。

## 2 新型分离材料在农产品质量安全分析中的应用

和传统的吸附剂相比,这些新型的纳米材料良好的吸附性能提高了分析方法的灵敏度,同时,适当的官能化提高了萃取选择性,结合一些高效的样品前处理技术,大大提高了分析方法的通量,在农产品质量安全分析中取得了很好的应用效果。

### 2.1 农药

农业生产中,农药的使用是保护农作物免受病虫害、提高农作物产量和品质的最重要手段之一。然而,农药不合理的使用可能会对农产品和环境产生安全问题,给人类的健康带来一定的威胁^[47,48]^,因此,开展农产品中农药残留量快速检测技术的研究具有非常重要的意义。一般来说,农药的种类繁多,农药多残留分析方法的开发是非常重要的。QuEChERS方法是目前应用最多的农药多残留分析方法,高性能的净化材料对于改善QuEChERS方法的性能具有重要的意义。李蕊岑等^[49]^开发了一种十八胺官能化的磁性纳米粒子介孔碳复合材料作为QuEChERS方法的吸附剂,结合GC-MS的分离检测,建立了一种同时测定绿叶菜中敌敌畏、毒死蜱、甲胺磷等16种有机磷农药残留量检测的样品前处理方法,该方法操作简单快捷,磁性介孔材料能很好地消除基质干扰并提高检测效率,检出限(LOD)为0.01~0.08 μg/mL,回收率为81.3%~94.0%,相对标准偏差(RSD)小于10%。Wang等^[50]^以支化聚乙烯亚胺和纳米级CaSO_4_官能化的多壁碳纳米管纳米复合物作为QuEChERS方法的吸附剂,克服了传统多壁碳纳米管由于强的*π-π*堆积作用导致平面结构的农药回收率低的问题,同时吸附剂对于去除蔬菜中的色素等基质干扰展现出极好的效果,通过吸附剂的重力沉降作用实现吸附剂与样品溶液的“自分离”,结合GC-MS/MS的分离检测,构建了一种果蔬中省时省力、便宜、灵敏、准确的农药多残留分析方法,农药在0.01 mg/kg和0.1 mg/kg水平下的加标回收率为75.3%~113.6%,检出限为0.0001~0.0026 mg/kg。Wang等^[51]^以L-丙氨酸修饰的MIL-88B为载体合成了磁性介孔材料,作为改进的QuEChERS方法的吸附剂,结合高效液相色谱-串联质谱同时测定了白菜、芹菜、豆角和大葱中的多种农药(灭多威、异丙威、克百威、3-羟基呋喃、乙草胺、吡虫啉),建立了一种简单、快捷、灵敏的蔬菜中6种农药残留量的分析方法,该方法具有极好的灵敏度(检出限为0.001~0.020 μg/kg)、良好的线性关系(*r*^2^≥0.9952)、较高的回收率(73.9%~107.7%)。我们课题组合成了一种磁性的二氧化锆吸附剂,将其作为改进的QuEChERS方法的吸附剂用于高脂类样品中农药残留的分析,通过吸附剂与脂肪酸类干扰物之间的路易斯酸碱相互作用,可以很好地除去基质中的干扰物,结合气相色谱-串联质谱的分离检测,建立了鱼肉和油料作物中农药多残留的分析方法^[52]^。

另一方面,设计合成对某一类农药具有特异性吸附作用的纳米材料也是近年来研究的热点问题。我们课题组^[53]^以花状Ni-NiO复合材料作为吸附剂,建立了一种磁性固相萃取结合LC-MS/MS快速、高选择性和高灵敏度的方法来检测食用植物油中的多菌灵(CBZ)和噻菌灵(TBZ),通过Ni^2+^与苯并咪唑类杀菌剂中供电子的咪唑基团之间的可逆相互作用,CBZ和TBZ被磁性花状Ni-NiO复合材料直接捕获,大大简化了样品制备的程序,该方法回收率为89.3%~110.7%,日内、日间精密度均小于10.9%,检出限为0.001~0.003 mg/kg。Yang等^[54]^制备一种基于MOFs材料NH_2_-MIL-125 (Ti)的滤纸吸附剂,通过等温吸附和吸附动力学分析发现,NH_2_-MIL-125 (Ti)滤纸膜对有机磷农药(OPPs)具有特异性的吸附能力,NH_2_-MIL-125 (Ti)基滤纸膜与OPPs之间的作用力主要是由*π-π*相互作用以及氨基和金属Ti对磷原子的亲和力产生的,对3种OPPs(亚胺硫磷、倍硫磷和杀螟硫磷)有快速萃取吸附的效果。表1列出了近3年来一些新型分离材料在农药中的应用。

**表 1:** 近年来一些纳米材料在农药残留分析中的应用

Nano-material	Analytes	Matrices	Detection method	Recoveries/ %		Adsorption mechanisms	LODs	Ref.
MNPCs	organophosphorus	fruit	GC-FPD	84.0	-116.0	π-π, hydrophobic	0.018-0.045 μg/L	[55]
MWCNTs	procymidone, atrazine, methidathion, etc.	cabbage apple and orange	GC-MS/MS	75.3	-113.6	π-π, hydrophobic	0.1-2.6 μg/kg	[56]
Magnetic porous carbon	methomyl, isoprocarb, carbofuran, etc.	honey	LC-MS/MS	61.6	-112	π-π, hydrophobic	0.5-25 μg/kg	[57]
AAS/NZVI/GO	methomyl, carbaryl, isoprocarb, etc.	sewage	LC-MS	93.4	-97.2	π-π, hydrophobic, electrostatic interaction	-	[58]
TP-MWCNTs	organophosphorus	vegetables	GC-MS	73.5	-114.2	π-π, hydrophobic interaction	0.81-7.63 μg/kg	[59]

MNPCs: magnetic nanoporous carbons; MWCNTs: multi-walled carbon nanotubes; AS/NZVI/GO: nano-zero-valent iron and graphene oxide; TP-MWCNTs: tetraethylenepentamine modified multi-walled carbon nanotubes; FPD: flame photometric detector; -: no data.

### 2.2 兽药

兽药广泛应用于畜牧业,用于预防和治疗动物疾病,促进动物生长,提高饲料转化率。然而,兽药残留很可能通过肉、蛋、奶等进入食物链^[60,61]^,从而对人体健康造成危害,包括过敏反应、抗生素耐药性、肠道微生物的变化等。因此,开展农产品中兽药残留的分析具有重要的意义。各种改性的磁性吸附剂在兽药残留分析中取得了很好的效果。Qiao等^[62]^制备了三维磁性花状SnS_2_复合材料Fe_3_O_4_@nSiO_2_-SnS_2_,将所制备的SnS_2_磁性复合材料用于牛奶蜂蜜中磺胺类抗生素(Sas)的富集,三维结构可以避免堆积和重新聚集,稳定性更好,有利于更多活性位点的暴露,SnS_2_材料与Sas之间存在疏水相互作用和静电相互作用,复合材料的制备简便易行,具有良好的化学稳定性,对环境更加友好,在优化条件下,该方法的检出限为0.025~0.250 ng/mL, 加标回收率为81.8%~119.7%,具有较好的重复性。Sahebi等^[63]^采用咪唑基离子液体修饰磁性壳聚糖纳米颗粒,将其作为磁性固相萃取的吸附剂萃取牛奶中22种抗生素及其代谢产物,离子液体和壳聚糖官能团上含有羟基、芳香环和季铵基团,可以与目标分子发生氢键、静电相互作用、疏水相互作用、*π-π*等相互作用,显著改善了萃取的选择性和萃取效率,结合液相色谱-串联质谱的分离检测,22种抗生素及其代谢产物的平均加标回收率为85.9%~107.5%,检出限可以达到0.04~0.19 μg/kg。

为了提高MOFs和COFs材料的吸附能力,Zhang等^[64]^利用天然的富含纤维素的玉米芯改性MOF@COF复合材料,合成了corncob@UiO-66-NH_2_@TpBD复合纳米材料,将其作为注射器固相萃取(IS-SPE)的吸附剂,用于鸡蛋、蜂蜜中磺胺类抗生素的萃取和分析,结果表明吸附剂与磺胺类抗生素之间存在*π-π*、氢键和配位等多重相互作用,结合超高效液相色谱-紫外检测,检出限为0.10~0.91 μg/L,回收率为81.2%~117.5%。Han等^[65]^制备了一系列稳定的层状多孔锆基金属有机骨架(HP-Nu-902-X),并将其用于磺胺类抗生素的富集,通过调节HP-Nu-902-X的孔尺寸和比表面积,大大提高了吸附性能,其中HP-Nu-902-80与目标化合物之间的*π-π*相互作用和氢键作用使其表现出良好的萃取效果,结合液相色谱检测,建立了从牛奶样品中提取Sas的高效分析方法,该方法的检出限为0.08~0.25 ng/mL,加标回收率为73.8%~100.5%。表2列出了近3年来一些新型纳米材料在兽药中的应用。

**表 2:** 近年来一些纳米材料在兽药残留分析中的应用

Nano-material	Analytes	Matrices	Detection method	Recoveries/ %		Adsorption mechanisms	LODs	Ref.
HP-Nu-902-X	sulfonamides	milk	HPLC-UV	73.8	-100.5	π-π, interaction	0.25 ng/mL	[65]
RAM-MMIPs	tetracyclines	egg	HPLC-UV	84.2	-96.5	electrostatic and hydrophobic interaction	2.21-2.67 μg/L	[66]
YS-Fe_3_O_4_@GC	sulfonamides	milk, meat	HPLC-UV	77.2	-118.0	π-π, electrostatic interaction	0.11-0.25 μg/L	[67]
Magnetic porous carbon	sulfonamides	milk	HPLC-DAD	76.9	-109.0	π-π, cation-π interaction, hydrogen bond	0.02-0.04 ng/mL	[68]
Fe_3_O_4_@CS-IL NPs	sulfonamides	milk	HPLC-MS/MS	85.9	-107.5	π-π, electrostatic interaction	0.04-0.19 μg/kg	[69]

HP-Nu-902-X: hierarchically porous zirconium-based metal-organic frameworks; RAM-MMIPs: restricted access media-magnetic molecularly imprinted polymers; YS-Fe_3_O_4_@GC: yolk-shell Fe_3_O_4_@graphitic carbon; Fe_3_O_4_@CS-IL NPs: ionic liquid-modified magnetic chitosan nanoparticles.

### 2.3 重金属

重金属污染已成为全球关注的重要问题,部分重金属可能具有致癌、致畸、致突变等危害,严重威胁到人类的健康和安全^[70]^,因此,准确分析农产品中重金属以保证农产品质量安全已变得越来越迫切。巯基能与某些金属离子发生强相互作用,可以作为高效的修饰试剂,为富集金属离子提供特定的官能团。Song等^[71]^合成了一种新型的2,5-二巯基-1,3,4-噻二唑(DMTZ)修饰的羟基磷酸铜@MOF复合材料DMP-Cu,并建立了以DMP-Cu为吸附剂结合原子荧光光谱联用的分散固相萃取技术选择性捕集大米样品中痕量总汞的检测方法。吸附机理研究表明,Hg^2+^的吸附过程符合准二级动力学和Langmuir吸附模型,在pH值为4时,吸附剂对Hg^2+^的最大吸附容量为249.5 mg/g, DMTZ的硫醇、含氮官能团与Hg^2+^的选择性强相互作用增强了其吸附过程,检出限为0.0125 ng/mL,相对标准偏差小于6%,回收率为98.8%~109%。

一些金属氧化物表面的路易斯酸碱位点也可以作为重金属离子的吸附位点。Yavuz等^[72]^合成了纳米海绵状Mn_3_O_4_,并首次将其用作旋涡辅助分离和预富集草莓、马铃薯、莴苣等农产品中铜和铅的新型吸附剂,结合火焰原子吸收光谱法检测食品和药品中的铜和铅含量,该方法简便、快速、可靠、准确,该方法的主要优点是预富集时间短,工作pH为酸性,吸附剂对铅的吸附容量大,该方法不需要使用有害的有机溶剂,在优化条件下,铜和铅的检出限为2.6 μg/L和3.0 μg/L。Alavinia等^[73]^首次合成了核壳结构的Fe_3_O_4_多巴胺纳米粒子,并将其用于涡流辅助磁分散固相萃取食品中的铜,采用火焰原子吸收光谱法检测。该方法操作简单,具有良好的准确度、精密度和选择性,在牛奶、麦片样品中的回收率良好,检出限为0.22 mg/L,吸附量为28 mg/g,表明该方法可成功应用于牛奶、麦片样品中铜的分离和预富集。表3列出了近3年来一些新型分离材料在重金属中的应用。

**表 3:** 近年来一些纳米材料在重金属分析中的应用

Nano-material	Analytes	Matrix	Detection method	Recoveries/ %		Adsorption mechanisms	LODs	Ref.
SAC-MNPs	Pb(Ⅱ)	red pepper	SQT-FAAS	102.6	-106.6	charge transfer	0.01-0.03 ng/mL	[74]
MMOF	Hg(Ⅱ)	shrimp	CVAAS	82.0	-112.0	electrostatic interaction	0.015 μg/kg	[75]
MnFe_2_O_4_-cellulose	Gd(Ⅱ)	lettuce	FAAS	95		charge transfer	0.5 μg/L	[76]
MWCNTs@MgAl_2_O_4_@TiO_2_	Pb(Ⅱ)	garlic	FAAS	91.0	-100.0	electrostatic interaction	0.42 μg/L	[77]
Fe_3_O_4_-Ti_3_AlC_2_	Cd(Ⅱ), Co(Ⅱ)	food	FAAS	92.0	-101.0	electrostatic interaction	0.093l, 0.297 μg/L	[78]

SAC-MNPs: stearic acid coated magnetic nanoparticle; MMOF: magnetic metal-organic framework; SQT-FAAS: slotted quartz tube flame atomic absorption spectrophotometry; CVAAS: cold-vapor atomic absorption spectrometry.

### 2.4 真菌毒素

真菌毒素是典型的农产品污染物,极易通过食物链在动物和人体内蓄积,到目前为止,已知有超过400种真菌毒素,主要由曲霉属、镰刀菌属、青霉属和交链孢属这四类真菌产生。真菌毒素对人和动物有致癌、致畸作用,对肝、肾、神经、免疫系统均有危害。近年来,各种新型前处理材料的开发有效提高了分析方法的准确性、灵敏度和响应速度,消除了农产品中基质的干扰,为真菌毒素的分析研究提供了新的思路和手段。其中,碳基纳米材料可以提供丰富的*π-π*相互作用位点,在真菌毒素分析中应用非常广泛。Xu等^[79]^采用化学共沉淀法和多巴胺原位氧化自聚法制备了PDA@Fe_3_O_4_-MWCNTs纳米材料,开发了一种新的、绿色、经济高效的磁性固相萃取法,用于从食用植物油样品中提取黄曲霉毒素和赭曲霉毒素。当磁性纳米粒子表面被可电离官能团修饰时,其表面的电荷通常会发生相应的变化,真菌毒素与PDA层和MWCNTs之间存在的*π*键、氢键以及一定的疏水相互作用加速了吸附过程。建立了磁性固相萃取结合高效液相色谱荧光检测器快速检测食用油中真菌毒素的方法,在最佳条件下对食用油中6种真菌毒素进行了分析,回收率为70.15%~89.25%。Yu等^[80]^采用溶剂热法合成了磁性石墨烯纳米复合材料Fe_3_O_4_/rGO,通过*π-π*堆积作用,成功地将该纳米复合材料用作磁性固相萃取吸附剂测定食用植物油中的黄曲霉毒素,结合高效液相色谱-柱后光化学衍生化-荧光检测,建立了食用油中黄曲霉毒素的简便、快速、高效、准确的分析方法,对黄曲霉毒素B_1_和B_2_的检出限分别为0.02 μg/kg和0.01 μg/kg,加标回收率为80.4%~106.0%。

利用金属氧化物表面羟基的特殊性质,也可将金属氧化物作为真菌毒素的吸附剂。Du等^[81]^将氧化锆纳米材料作为分散固相萃取的吸附剂,用于农产品中黄曲霉毒素、伏马毒素、赭曲霉素等多种真菌毒素的萃取,氧化锆纳米材料表面含有丰富的羟基官能团,可以通过氢键、离子相互作用等吸附真菌毒素类污染物,结合超高效液相色谱-四极杆飞行时间质谱的分析,真菌毒素的检出限可达0.0022~0.033 μg/kg,回收率为84.27%~104.96%。

MOF和COF吸附剂由于其多孔的结构、丰富的*π-π*相互作用位点也经常用于农产品中真菌毒素类污染物的萃取富集。Li等^[82]^以1,2,4,5-四-(4-甲酰基苯基)苯(TFPB)和对苯二胺(PPD)为原料,通过一种简便的方法设计并合成了一种新型的磁性COF纳米材料,首次使用磁性COF作为磁分散固相萃取(MDSPE)的吸附剂来富集食品基质中的黄曲霉毒素,黄曲霉毒素与Fe_3_O_4_@COF表面的氨基与乙醚形成氢键,COF层中的苯环与黄曲霉毒素中的苯环或共轭双键之间可能存在*π*相互作用和疏水相互作用,这在一定程度上促进了黄曲霉毒素的吸附,对AFs的吸附容量为69.5~92.2 mg/g,加标回收率为76.4%~112.5%,日内和日间精密度均小于15%。

金属氧化物化学性质稳定,对其进行适当的官能化,可以显著改善吸附剂的萃取性能。Zhu等^[83]^建立了一种基于离子溶液功能化氧化锌纳米花(ILs@ZnO NFs)的简单方法,结合分散微固相萃取(D-μSPE)技术提取小麦和花生样品中4种黄曲霉毒素(AFB_2_、AFG_2_、AFB_1_和AFG_1_),因其ILs@ZnO NFs具有咪唑基团,这些咪唑基团可以作为与AFs内脂环上羰基进行静电相互作用的作用位点,在最佳实验条件下,该方法具有良好的线性关系,相关系数高(≥0.994), LOD为0.024~0.067 μg/kg,回收率为93.8%~105.1%。表4列出了近3年来一些新型分离材料在真菌毒素中的应用。

**表 4:** 近年来一些纳米材料在真菌毒素中的应用

Nano-material	Analytes	Matrices	Detection method	Recoveries/ %	Adsorption mechanism	LODs	Ref.
Novel α-Fe_2_O_3_ nanocubes	DON, AFB_1_	mung	-	94.4-128.0, 93.3-128.3	electrostatic interaction	0.18 ng/mL, 0.01 ng/mL	[84]
MGO/MOF-808@ MIP	AFBs	rice	LC-MS/MS	-	π-π, hydrogen bonds	0.09 ng/mL	[85]
PEG-MWCNTs-MNP	AFB_1_	milk	UHPLC-Q-Exactive HRMS	81.8-106.4	π-π, hydrophobic, hydrogen bonds	0.005-0.050 μg/kg	[86]
MWCNTs-Fe_3_O_4_	AFB_1_	grain	UPLC-MS/MS	73.5-112.9	π-π, hydrophobic	0.0021-5.4457 μg/kg	[87]
MOF-235	AFBs	vegetable oils	UPLC-MS/MS	90.1-99.2	π-π	-	[88]

MGO/MOF-808@MIP: surface-imprinted Zr metal-organic framework on the magnetic graphene oxide; PEG-MWCNTs-MNP: polyethylene glycol-multiwalled carbon nanotubes magnetic nanoparticles; MWCNTs-Fe_3_O_4_: magnetic (Fe_3_O_4_) nanoparticles modified with multiwalled carbon nanotubes; MOF-235: metal organic frameworks.

### 2.5 其他污染物

环境中的污染物如环境激素、多环芳烃以及食品添加剂也对农产品质量安全产生了较大威胁。环境激素是一类外源性化合物,又被称作环境荷尔蒙或内分泌干扰物(EDCs),这些化合物可通过其在食物链中的迁移和转化并不断累积,干扰人体正常的激素分泌,导致人体发育和生殖异常,对人类健康产生严重危害。多环芳烃为一种持久性有机污染物,具有致癌、致畸、致突变作用,并能够持久存在于环境中。食品添加剂可以改善食品色、香、味,还可以起到防腐和保鲜的作用,但食品添加剂使用不当,包括使用非法添加剂、超量使用食品添加剂、使用伪劣食品添加剂均会对人体健康造成严重危害。

很多新型的纳米材料用于农产品中环境污染物的萃取净化。Belenguer-Sapiña等^[89]^合成了一种以环糊精为表面改性剂的UVM-7型介孔二氧化硅吸附剂,该材料含有环糊精单元,可有效改善吸附剂的选择性,用于从瓶装苹果汁中提取内分泌干扰化学物质,回收率为94%~100%,日内和日间精密度均在6.8%以内。Liu等^[90]^提出了一种制备硼酸功能化核壳结构磁性COF纳米复合材料的简单方法,即以Fe_3_O_4_纳米粒子为磁核、硼酸功能化的COFs为壳所合成的纳米复合材料Fe_3_O_4_@COF@BA显示出大的比表面积、高的磁响应性以及理想的化学和热稳定性,结合高效液相色谱-串联质谱法,用于肉类样品中内分泌干扰物的磁性固相萃取,在牛肉、鸡肉和猪肉样品中内分泌干扰物的回收率为88.8%~104.2%,检出限为0.08~0.72 μg/kg,RSD低于5.4%。Peng等^[91]^合成了磁性介孔二硫化钼/石墨纳米片(mm-MoS_2_/GN),并将其应用于食用油样品中13种多环芳烃的磁固相萃取,mm-MoS_2_/GN多孔蓬松结构,具有丰富的活性位点(*π*相互作用、静电、氢键)和两亲性,是分离和富集食用油中痕量多环芳烃的理想材料,在最佳MSPE和GC-MS条件下,该方法的线性范围为1.0~100.0 μg/kg, 13种多环芳的检出限为0.10~2.50 μg/kg,日内和日间精密度分别小于3.0%和3.6%,加标回收率为70.2%~112.6%,是一种新颖、快速、经济、有效的多环芳烃检测方法。该方法成功应用于国内市场上多环芳烃样品的检测。Liu等^[92]^将1,6-二(4-甲酰苯基)-3,8-二((4-氨基苯基)乙基)芘(BFBAEPy)自聚合并包覆在改性Fe_3_O_4_表面,合成了一种具有核壳结构的磁性共价有机骨架(Fe_3_O_4_@COF)纳米复合材料,Fe_3_O_4_@COF具有比表面积大、孔径合适、静电相互作用和*π-π*叠加相互作用等优点,是一种高效的MSPE吸附剂,用于辣椒粉和花椒中非法添加的罗丹明B(RhB)的富集检测,回收率高(91.7%~97.5%),重复性好(RSD<3.8%), LOD低至0.0038 μg/mL,对农产品中RhB的检测表现出良好的应用前景。表5列出了近3年来一些新型分离材料在其他污染物中的应用。

**表 5:** 近年来一些纳米材料在其他污染物分析中的应用

Nano-material	Analytes	Matrices	Detection method	Recoveries/ %		Adsorption mechanisms	LODs	Ref.
rGO/ZnFe_2_O_4_	estrogens	fish	HPLC-DAD	73.5	-104.1	π-π, hydrogen bonds	0.01-0.02 ng/mL	[93]
Fe_3_O_4_/GO	melamine	dairy products	HPLC-UV	97.2	-103.1	π-π, electrostatic interaction	0.03 μg/L	[94]
MWCNT-MNP	PAHs	milk	GC-MS	86.1	-100.3	π-π	0.040-0.075 μg/kg	[95]
NH_2_-Zn/Fe-MIL-8	phytohormones	vegetable	HPLC	82.6	-98.1	π-π, hydrogen bonds	0.07-0.15 ng/mL	[96]
nano-g-C_3_N_4_/UiO- 66-NH_2_	carmine, brilliant blue	blueberry	HPLC	82.6	-105.8	electrostatic interaction	0.08-0.8 ng/mL	[97]

rGO/ZnFe_2_O_4_: reduced graphene oxide/ZnFe_2_O_4_ nanocomposite; Fe_3_O_4_/GO: magnetite/graphene oxide nanocomposite; MWCNT-MNP: MWCNTs magnetic adsorbent; NH_2_-Zn/Fe-MIL-8: amino-modified Zn/Fe bimetallic metal-organic frameworks; nano-g-C_3_N_4_/UiO-66-NH_2_: nano-graphitic carbon nitride and three-dimensional MOF; PAHs: polycyclic aromatic hydrocarbons.

## 3 总结与展望

农产品中农药、兽药、重金属、真菌毒素等污染物快速、高效、灵敏的分析对于保障农产品质量安全具有重要的意义。发展高效的固相萃取吸附剂,实现农产品中污染物快速的富集和净化,是农产品质量安全分析准确性的关键手段之一。本文综述了近年来农产品质量安全分析中应用较多的功能材料,如磁性材料、碳基材料、金属和金属氧化物材料、金属有机骨架材料和有机共价骨架材料等,介绍了材料的吸附特征,对它们在农产品质量安全分析中的应用进行了综述。从目前报道的结果来看,大部分新型材料具有良好的孔结构和大的比表面积,稳定性好,可根据需要对其结构和功能团进行设计改性,在农产品质量安全分析中取得了很好的应用效果。

当前,农产品污染物分析的前处理方法向着绿色、高选择性、快速和低成本的方向发展,这对样品分析过程的前处理材料提出了更高的要求。通过使用少量的吸附剂材料和样品量的小型化分析技术,减少试剂的用量,可以实现绿色萃取,而较少量的吸附剂意味着较低的吸附容量,因此发展多孔的大比表面积的吸附材料仍然是今后吸附剂材料发展的一个主要方向。另一方面,农产品基质复杂,提高吸附剂的选择性对于除去样品中的基质干扰非常重要,因此在保持高吸附容量的同时提高吸附剂的选择性,研究吸附机理,减少非特异性的基质干扰,对于提高分析方法的准确度和稳定性是非常重要的。此外,目前的样品前处理技术大部分是离线的,研究新型吸附材料与在线前处理方法相结合的技术,提高样品分析的通量,可以满足大批量农产品快速分析的需求。最后,前处理方法的成本是其能否推广应用的关键,要减少前处理分析方法的成本、开发高效吸附剂材料的大规模低成本的生产工艺是非常重要的。
